# Brain organoids and insights on human evolution

**DOI:** 10.12688/f1000research.18495.1

**Published:** 2019-05-30

**Authors:** Alysson R. Muotri

**Affiliations:** 1 Department of Pediatrics/Rady Children's Hospital San Diego, University of California San Diego, School of Medicine, La Jolla, CA, USA; 2 Department of Cellular & Molecular Medicine, University of California San Diego, School of Medicine, La Jolla, CA, USA; 3 UCSD Stem Cell Programme, University of California San Diego, School of Medicine, La Jolla, CA, USA; 4 Center for Academic Research and Training in Anthropogeny (CARTA), La Jolla, CA, USA; 5 Kavli Institute for Brain and Mind, University of California San Diego, School of Medicine, La Jolla, CA, USA

**Keywords:** brain organoids, evolution, pluripotent stem cells

## Abstract

Human brain organoids, generated from pluripotent stem cells, have emerged as a promising technique for modeling early stages of human neurodevelopment in controlled laboratory conditions. Although the applications for disease modeling in a dish have become routine, the use of these brain organoids as evolutionary tools is only now getting momentum. Here, we will review the current state of the art on the use of brain organoids from different species and the molecular and cellular insights generated from these studies. Besides, we will discuss how this model might be beneficial for human health and the limitations and future perspectives of this technology.

## Introduction

What is it that makes us uniquely human? Among several features of our species, perhaps the most impactful characteristic consists of our sophisticated brains and all the abilities and advanced lifestyles we all enjoy. However, a complex brain came with a cost. The vulnerability for human-specific neurological disorders might well be an undesired evolutionary trade-off. Although many genetic mutations might also cause diseases in other animals, the genomic landscape of humans makes our species more susceptible to certain neurological disorders ^1, 2^. Gaining a clearer understanding of human brain evolution is crucial to interpreting how human genetic variants lead to disease. Therefore, understanding the evolutionary path of the modern human brain will likely illuminate the origins of conditions such as autism, dementia, or schizophrenia, which are considerable burdens to all present and future human societies.

Our knowledge of human brain development is deficient and there are gaps in the critical steps leading to the cellular organization and the formation of functional networks that are the basis of cognition. The limited accessibility of the human (and also non-human primate, or NHP) brain has blocked our understanding of neurodevelopment, especially at very early stages. During embryogenesis, a limited number of pluripotent embryonic stem cells will give rise to a multi-cellular and complex nervous system in the brain, which orchestrates many of the autonomous and non-autonomous functions of the body. *In utero* experimental access to ape brains is not always possible. Instead, scientists have relied on less invasive passive alternatives, such as ultrasound and functional imaging. Most of our understanding of normal and pathological brain conditions comes from postmortem tissues that represent only a blurry snapshot of a highly dynamic tissue. Also, the neuroanatomical and functional heterogeneity that individuals have because of their genetic and environmental backgrounds adds another obstacle to fully understanding the typical and unhealthy progression of neurodevelopment ^3^.

Brain organoids, generated from pluripotent stem cells, are multi-cellular, three-dimensional self-assemble miniaturized structures that mimic the dynamic organization ^4, 5^ and molecular profile of the developing human embryonic and fetal brain ^6– 9^. These structures can grow free floating in media or embedded in matrigel and develop different brain regions using endogenous patterning cues ^10, 11^. Alternatively, cells could be patterned early on by exposure to specific cocktails of factors, coaxing the identity of the cells toward a specific brain region ^4, 12, 13^. Differently patterned organoids independently created could be fused, exchanging signals among the different brain regions, stimulating cell exchange ^14– 16^ or reciprocal projections ^12, 17^. The “lego-organoid” approach can recreate specific circuit formations in a more controlled and reproducible fashion than non-patterned cerebral organoids.

Human brain organoid has recently contributed to the understanding of several neurological conditions (reviewed extensively in 18– 20), including typically human conditions such as schizophrenia ^21^ and autism ^22^. Interestingly, the fact that brain organoids can be generated from induced pluripotent stem cells (iPSCs) ^23, 24^, reprogrammed from somatic cells of any species, offered an unprecedented opportunity to compare early stages of human neurodevelopment with those of other primates, including our closest NHP relatives: the chimpanzees ( *Pan troglodytes*) and bonobos ( *Pan paniscus*) ^25– 27^. These three species have very similar genomes, including nearly 98% of alignable genomic sequence ^28^. However, cellular and molecular phenotypes, especially at similar stages of development, are difficult to establish, mainly owing to limited access to live embryonic material from humans and NHPs. Thus, the study and manipulation of brain organoids in a dish are novel and promising evolutionary tools ^25^.

## LINEs might be responsible for human-specific diseases

The first comparative study between human and NHP iPSCs revealed unexpected differential regulation of long interspersed element-1 (L1, also known as LINE-1) endogenous retrotransposons ^29^. L1 elements are autonomous mobile elements present in around 20% of the mammalian genomes and have remained active during evolution ^30– 33^. Mobilization of L1s can impact the human genome and is associated with several human disorders ^34, 35^. Among the top 50 genes that were differentially expressed between human and NHP iPSCs, two L1-restricting factors—APOBEC3B (also known as A3B) and PIWIL2—were found upregulated in human cells. The experimental manipulation of A3B and PIWIL2 levels in the pluripotent stem cells supported a causal inverse relationship with L1 retrotransposition. Increased levels of L1 retrotransposition suggested that NHP-derived iPSCs would be more susceptible or permissive to new insertions. An increased copy number of species-specific L1 elements in the genome of chimpanzees was observed when compared with humans. The data support the idea that increased L1 mobility in NHPs might not be limited to the pluripotent stage and may also occur in the germline during primate evolution. Thus, it is possible to speculate that the activity of L1 elements has differentially shaped the human genome and still has adaptive significance.

The RNA from L1 elements might also have an impact on human health. Aicardi–Goutières syndrome (AGS) type I is characterized by a dramatic neuronal loss, leading to lifelong disability ^36^. AGS can be caused by mutations in the three-prime repair exonuclease I ( *TREX1*) gene ^37^. Curiously, rodent models of AGS do not mimic the severe neurological aspect of the human condition ^38^. However, AGS-derived brain organoids do mimic the neurodegeneration and striking microcephaly seen during patient neurodevelopment *in utero* ^39^. Neuronal death in the brain organoids was caused, at least partially, by a substantial exposure of type I interferon, a pro-inflammatory cytokine secreted by astrocytes. The innate immune reaction in the astrocytes was triggered by the accumulation of L1 retrotransposons in the cell because of TREX1 absence. L1 elements are different between humans and other animals ^40^. Evidence that L1 activity could contribute to other neurological disorders, such as Huntington’s disease ^41^, Down syndrome, and Alzheimer’s disease ^42^, and aging ^43^ has recently been provided. It is an attractive hypothesis that the sequence-specific L1 differences might contribute to several human conditions, creating the potential for unconventional treatments using reverse transcriptase inhibitors, such as anti-HIV drugs.

## Emergent viruses and brain defects

Endogenous viruses are not the only ones affecting human evolution. Human brain organoids played an essential role in the causal link demonstration between the Brazilian Zika virus and the microcephaly outbreak in Brazil. The relatively slow human neurodevelopment, compared with that of other experimental animal models mimicked by the human brain organoids in a dish, allowed investigators to dissect how the virus could infect neural progenitor cells leading to defects in the cortical plate ^44– 46^. Moreover, brain organoids from NHPs revealed differences in viral replication rates of the Brazilian strain compared with the African Zika virus at the same developmental stage. This observation might suggest that the Zika virus can adapt to different primates. Evidence for specific mutations in the circulating Brazilian Zika virus has emerged ^47^ but it is unclear whether these variants were responsible for the dramatic phenotypes seen in the affected human babies. The abundance of human brain organoids was also a positive feature to rapidly screen for drugs that could prevent infection ^48, 49^ or block viral replication and eventual vertical transmission ^50^.

## Exploring cortical development

In the past, testing hypotheses about human brain evolution was restricted to manipulations in animals or non-relevant cell types. Owing to cellular reprogramming, it is now possible to compare differences and similarities between human neurodevelopment and that of other primates without the use of embryonic materials that are ethically and technically difficult to access ^29, 51^. The ability to create cortical brain organoids from human and other NHP iPSCs provides a unique opportunity to study the expansion of the neocortex in a dish ^26, 52^. By contrasting brain organoids of humans with those of chimpanzees, it was possible to determine a subtle differentiation between timing and lengthening of prometaphase-metaphase in human apical mitosis that is specific to proliferating neural progenitor cells ^27, 53, 54^. It is possible that subtle differences at very early stages have a more dramatic impact later in development and thus set humans apart from other primates.

Cortical tissues generated from human, chimpanzee, orangutan, and rhesus iPSC-derived brain organoids were also used for a dynamic transcriptional analysis ^55^. Several long non-coding RNAs (lncRNAs) were detected in specific cell types and stages of differentiation in all of the species. LncRNAs are implicated in several molecular mechanisms, including the regulation of neurodevelopment ^56, 57^. The conservation of pattern expression on these tissue-specific lncRNAs in all of these primates indicates a possible role in transition stages during neurodevelopment. Another recent work that contrasted brain organoids from human and NHP iPSCs also described gene network conservation among primates while identifying a set of 261 genes that are human-specific ^58^. Some of these genes overlap with recent chromosomal segmental duplications ^59^. Interestingly, increased activation of the PI3K/AKT/mTOR pathway was validated in the radial glia cells of the outer subventricular zone of human fetal brain tissues, possibly contributing to the neocortex expansion during evolution. The creation of a “brain organoid zoo”, which has representatives of different primates and other species, might help us to resolve critical molecular and cellular steps to comprehend brain evolution better.

## Discussion and future perspectives

Our natural interest in understanding the processes that make us human goes beyond mere anthropocentrism and philosophical debates about the human condition. Novel knowledge gained from interspecies comparisons can potentially contribute to biomedical advances. For example, humans and other primates can be distinguished by AIDS progression ^60^, malaria vulnerability (immunity against *Plasmodium falciparum*) ^61^, Alzheimer’s disease (absence of neurofibrillary tangles) ^62^, and susceptibility to certain cancers ^63^ as well as other differences ^64, 65^. The identification and characterization of the cellular and molecular mechanism that distinguishes humans from our closest relatives at early stages of embryogenesis and in specific types of cells are likely to become a new resource for evolutionary studies ^66^. In this context, recent advances in iPSC-derived neural progenitor cells and brain organoids are an attractive tool to dissect cellular and molecular events that contribute to the uniqueness of the human brain. However, this artificial *in vitro* approach is not without serious inherent limitations. Most comparative studies are standardizing the growth of the brain organoids using human culture conditions. This “humanized” situation is likely masking important differences among species. The use of “neutral backgrounds”, such as transplanting different primate cells in the mouse brain ^54^, could mitigate this concern. Other limitations are intrinsic to the brain organoid model: lack of specific cell types, cellular stress, organized brain regions, and endogenous vascularization. The use of patterned brain organoids might help to reduce experimental variability and increase confidence in the data, especially when differences are subtle. Thus, owing to these limitations, validation or confirmation of findings in primary tissues ^58, 67^ or even intact functional brain ^68^ is the gold standard in this field.

Nonetheless, it is expected that some of these technical shortcomings might be resolved in the next few years (reviewed in 18, 69). In the future, evolutionary studies using brain organoids would benefit from genome editing for candidate approaches. More sophisticated comparisons will also incorporate functional readouts, such as the emergence of network activity and oscillatory waves in long-term, mature brain organoids ^68^. Finally, the perspective to extrapolate the comparative approach of modern humans to other extinct hominins, such as Neanderthals and Denisovans, by using human brain organoids carrying ancestral genetic variants will lead to an entire new field ^70^.

## Abbreviations

AGS, Aicardi–Goutières syndrome; iPSC, induced pluripotent stem cell; L1 or LINE-1, long interspersed element-1; lncRNA, long non-coding RNA; NHP, non-human primate; TREX1, three-prime repair exonuclease I
